# Preclinical Models of Brain Metastases in Breast Cancer

**DOI:** 10.3390/biomedicines10030667

**Published:** 2022-03-13

**Authors:** Natasha N. Knier, Sierra Pellizzari, Jiangbing Zhou, Paula J. Foster, Armen Parsyan

**Affiliations:** 1 Department of Medical Biophysics, Western University, London, ON N6A 5C1, Canada; nknier@uwo.ca (N.N.K.); pfoster@robarts.ca (P.J.F.); 2 Imaging Laboratories, Robarts Research Institute, London, ON N6A 5B7, Canada; 3 Department of Anatomy and Cell Biology, Western University, London, ON N6A 3K7, Canada; spelliz2@uwo.ca; 4 Department of Neurosurgery, Yale University, New Haven, CT 06510, USA; jiangbing.zhou@yale.edu; 5 London Regional Cancer Program, London Health Science Centre, London, ON N6A 5W9, Canada; 6 Department of Oncology, Western University, London, ON N6A 4L6, Canada; 7 Department of Surgery, Western University, London, ON N6A 3K7, Canada

**Keywords:** breast cancer, brain metastasis, preclinical animal models, patient-derived xenografts, animal imaging, multimodal imaging

## Abstract

Breast cancer remains a leading cause of mortality among women worldwide. Brain metastases confer extremely poor prognosis due to a lack of understanding of their specific biology, unique physiologic and anatomic features of the brain, and limited treatment strategies. A major roadblock in advancing the treatment of breast cancer brain metastases (BCBM) is the scarcity of representative experimental preclinical models. Current models are predominantly based on the use of animal xenograft models with immortalized breast cancer cell lines that poorly capture the disease’s heterogeneity. Recent years have witnessed the development of patient-derived in vitro and in vivo breast cancer culturing systems that more closely recapitulate the biology from individual patients. These advances led to the development of modern patient-tissue-based experimental models for BCBM. The success of preclinical models is also based on the imaging technologies used to detect metastases. Advances in animal brain imaging, including cellular MRI and multimodality imaging, allow sensitive and specific detection of brain metastases and monitoring treatment responses. These imaging technologies, together with novel translational breast cancer models based on patient-derived cancer tissues, represent a unique opportunity to advance our understanding of brain metastases biology and develop novel treatment approaches. This review discusses the state-of-the-art knowledge in preclinical models of this disease.

## 1. Background

Breast cancer (BC) is the second leading cause of cancer-related deaths among women. Considerable progress has been made towards understanding the biology of BC, leading to the development of effective treatments. However, metastatic BC still confers a poor 5-year relative survival of ~26% [1,2]. Up to 30% of BC metastases occur in the brain, with a risk of death within a year reaching 62% [3,4,5]. The most common sites of BC brain metastases (BCBM) include the frontal lobe, cerebellum, and to a lesser extent, the brain stem [6]. A substantial decrease in the quality of life is observed in breast cancer patients with brain metastases due to the neurological sequalae of the disease. 

Since BC is a molecularly heterogeneous disease, some of its molecular variants exhibit higher rates of brain metastases, such as those that do not express estrogen (ER) and progesterone (PR) receptors but are positive for the human epidermal growth factor receptor 2 (HER2). These HER2+ subtypes (that account for up to 15% of all BC) tend to metastasize to the brain at a higher (~50%) rate and lead to ~6 months median survival [7,8,9]. While the reason for this increased metastatic potential for HER2+ cancer is likely multifactorial, it has been posited that the driving factors of BCBM in this subtype may be attributed to interactions between HER2 and other receptors, including epidermal growth factor receptor (EGFR) and HER3 [10]. Treating BCBM has been particularly challenging due to the unique anatomical and functional features in the brain. Novel therapies are being developed to improve systemic control, however, poor drug penetration of the blood–brain barrier (BBB) can create a sanctuary for tumor cells in the brain during treatment and lead to an increased incidence of BCBM. Furthermore, dormancy observed in some BCBM may hamper the effects of chemotherapy and serve as a factor for recurrence that can occur decades after what was thought to be a successful treatment [11]. The poor outcomes and failures of treatment of BCBM are also a reflection of the differences in the biology of brain metastases compared to that of the early stages of BC [2,12]. These differences remain poorly understood. Hence, understanding the BCBM biology and subsequently testing novel therapeutic modalities that account for unique anatomic features of BCBM is of clinical importance [13]. Success in developing effective treatments for BCBM is founded on the availability of preclinical experimental models that effectively recapitulate BCBM in patients and sensitive experimental detection methods. This review discusses current knowledge related to preclinical BCBM models and their detection methods. 

## 2. Models of Breast Cancer Brain Metastases

Preclinical experimental models for BCBM, ideally, should closely represent a milieu in which metastases develop, capture the heterogeneity of BC [14], and incorporate brain anatomical nuances, such as the BBB. The latter can cause BCBM to become inaccessible to drugs and, hence, is an essential consideration for drug testing [15]. Although in vitro microfluidics and ex vivo coculture models have been described to incorporate the BBB [16,17], stromal cell interactions, and BCBM infiltration patterns [18], in vivo animal models remain the benchmark for preclinical models [19] (Table 1). 

**Figure 1:**
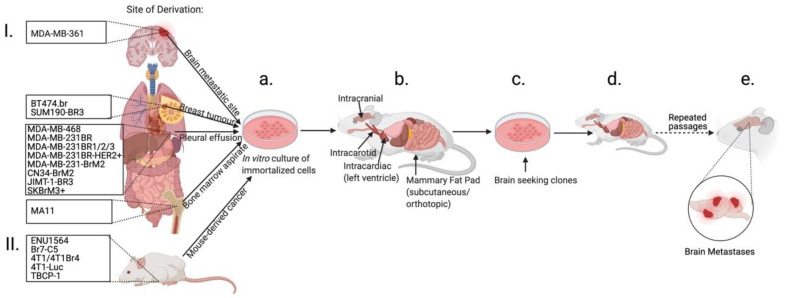
Establishment of breast cancer brain metastasis from immortalized breast cancer cell lines. (**I**) Immortalized breast cancer cell lines are established from breast cancer primary tumors or metastases or (**II**) spontaneously developed breast cancer in the mouse model. Cells are then cultured in vitro (**a**) and introduced into mice (**b**) with the goal of developing brain metastasis. In some models, formed brain metastases are then dissociated to single cells and passaged in vitro (**c**) to generate a brain-seeking clone and then are reintroduced into the animal (**d**). Often multiple re-passaging cycles are used to establish brain-seeking clones until an efficient BCBM mouse model is generated (**e**) (see text). Created with BioRender.com.

**Immortalized Human Breast Cancer Cell Line Models.** Various xenogeneic models of BCBM in immunocompromised mice have been described (Table 1) based on human immortalized breast cancer cell (IBCC) lines. Some of the initially described MDA-MB-361 [21] and MDA-MB-468 cell line-based models used intracarotid injections in mice to study treatment responses [68], the BCBM microenvironment [69], and BBB impairment in response to BCBM [70]. Those models, however, had poor selectivity for the formation of metastases in the brain. Hence, attempts to establish models that preferentially form metastases in the brain have been made (Figure 1) through the selection of cell populations that have a propensity to form BCBM, such as mucin (MUC1) secreting MA11 cell line derivatives [24], that after intracardiac injections in BALB/c nu/nu mice preferentially formed BCBM in 87% of animals. 

**Figure 2:**
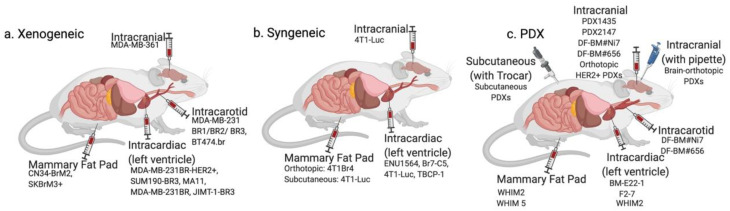
Methods of introduction of cancer cells into an experimental animal to generate breast cancer brain metastatic models. Various introduction methods applied for (**a**) xenogeneic models, (**b**) syngeneic models, and (**c**) patient-derived xenograft models are presented (see text). Breast cancer cells are most commonly introduced into mice via intracranial, intracarotid, intracardiac, or mammary fat pad injections or implantation. More sophisticated approaches, such as ligation of the external and common carotid arteries during intracarotid injection, intracranial transplantation using pipette tip through burr hole, and bilateral subcutaneous injection using a trocar have also been described for PDX models. Created with BioRender.com.

The most common approaches to increase the efficiency of BCBM formation are based on clonal selection of cell populations from parental IBCC lines that have a propensity to form brain metastases (Figure 2). To establish such a brain-seeking clone, a parental ER/PR/HER2-, or triple negative BC (TNBC), MDA-MB-231 cell line was injected intracardially into nude mice and after 3–4 weeks [39], cells from brain metastases were cultured in vitro and re-inoculated into mice. This procedure was repeated six times until the brain-seeking MDA-MB-231BR (231BR) cell line was established, resulting in 100% frequency of metastases to the brain and no metastases to other organs. Additional subclones of the 231BR cell line have been developed by performing three rounds of selection and intracarotid injections in mice, resulting in the BR1, BR2, and BR3 sublines. These sublines varied from the original 231BR cells in that they expressed elevated levels of VEGF-A (vascular endothelial growth factor A), which has been shown to be critical in the development of BCBM [71]. Indeed, they led to the shorter survival of mice and development of more brain metastases compared to the 231BR cells [40]. The MDA-MB-231-BrM2 subline has been established using a similar approach of intracardiac injections and clonal selection through an additional round of in vitro and in vivo culturing and led to metastases in the cerebrum, cerebellum, brainstem, and leptomeninges [43]. Using a similar methodology but a different TNBC cell line, CN34, a CN34-BrM2 clone was described, that after intracardiac or mammary fat pad injections, metastasized to the same locations in the mouse brain [43].

Given that HER2+, compared to HER2-, has a higher propensity to metastasize to the brain [7,8], various approaches have been used to establish HER2+ models. An MDA-MB-231BR-HER2+ (231BR-HER2+) line was developed by transducing 231BR TNBC cells with enhanced green fluorescent protein (eGFP), and then transfecting with HER2 cDNA [48]. When compared to 231BR cells after intracardiac injection in BALB/c nude mice, 231BR-HER2+ developed BCBM more aggressively with an increased number of large metastatic tumors. Other HER2+ brain-seeking sublines based on JIMT-1, SUM190, and BT474 lines have been described. JIMT-1-BR3 was established [41] through intracardiac injections of NCr-nu/nu mice and culturing of removed metastases in vitro with reintroduction in vivo two additional times. JIMT-1-BR3 formed brain metastases in 100% of mice. A similar approach was utilized to establish the SUM190-BR3 HER2+ line [49]. BT474.br was established through right carotid injections of BT474 cells to select for brain-seeking cells in vivo [29]. After 3 months, overt brain metastases with a high HER2 expression level formed predominantly in the right hemisphere and micrometastases in the left hemisphere. These were selected in vivo through 2–3 rounds of intracarotid injection to create the Br.2 and Br.3 sublines to hyperactivate Src (family of non-receptor tyrosine kinases). A combination regimen using a Src-targeting Saracatinib with lapatinib (targets EGFR and HER2) prevented the growth of disseminated cancer cells by causing cell cycle arrest. Additionally, a combination treatment of neratinib and the c-MET inhibitor cabozantinib was tested in a model where brain-seeking SKBrM3 cells expressing high levels of c-MET and the EGFR were selected for markers of invasiveness (vimentin and ZEB1) to establish a brain-seeking SKBrM3+ subline [53]. The latter was injected into the mammary fat pad of athymic nude mice and tumor growth was monitored with bioluminescence imaging (BLI). High incidences of brain and other organ metastases were observed using this subline and their occurrence and proliferation were inhibited by a combination treatment. 

**Syngeneic Models.** To address the shortcomings associated with the absence of the immune components in BCBM in xenogeneic models, syngeneic models have been studied (Table 1). These models have important utility given the development of novel immunotherapeutics and their introduction into clinical practice for the treatment of metastatic BC [72]. A brain-seeking clone of the ENU1564 rat mammary adenocarcinoma cell line, Br7-C5, was established, using intracarotid injections to a rat and further selection through in vitro re-culturing of brain metastases and in vivo reinoculation, leading to consistent, however nonexclusive, metastases to the brain [54]. A brain metastatic subline (4T1BM), a derivative of 4T1 cells, was established by orthotopic implantation to develop BCBM, but resulted in poor brain metastatic development [55]. However, after four rounds of selection in vitro and in vivo via inoculation into a BALB/c mouse mammary gland, a 4T1Br4 subline was developed that metastasized to the brain, with a higher incidence (20%) than the parental 4T1 cell line (7%) [57]. A 4T1 cell-based model using either intracranial or intracardiac injection of luciferase-transduced 4T1 cells into mice has also been described, which resulted in higher (compared to subcutaneous injection) rates (25%) of BCBM as assessed by BLI [59]. The TBCP-1 HER2+ cell line, established through clonal selection for low ER/PR and high HER2 expression to study neratinib, an irreversible pan-HER inhibitor, resulted in metastases formation in 80% and 60% of mice when injected into the heart compared to the 4th inguinal fat pad, respectively [60]. The study found that neratinib inhibits tumor growth and brain metastasis, conferring a significant increase in disease-free survival [60]. 

**Patient-Derived Models.** To better capture disease heterogeneity and patient treatment responses, patient tissue-derived models, such as patient-derived xenografts (PDX), have been developed. These systems serve as a basis for the next-generation of preclinical translational research and personalized medicine [73,74,75]. Therefore, there has been growing interest in applying these models to studies of BCBM (Table 1). Generation of metastases by PDX models (Figure 2) is relatively challenging. Orthotopic PDX in the mammary fat pad of NSG mice has been described where 1/7 models developed brain metastases that represented <2% of overall metastases [76]. However, low rates of brain metastasis reported in this study could be partially attributed to the use of an orthotopic model and to the histological analysis for brain metastasis that could fail to detect small tumor deposits. Another TNBC PDX from patient brain metastases, F2-7, in NSG mice has been described [61], where the xenograft tissue was dissociated to a single cell suspension and labeled with luciferase in an in vitro culture. Subsequently, labeled cells were injected into the mammary fat pad of the mice and BLI was used to monitor the presence of metastases and their growth. 

In order to increase rates of BCBM, investigators used intracarotid, intracardiac, and intracranial injections. In the BM-E22-1 TNBC model, tumor tissue was propagated through implantation into the mammary fat pad of NSG mice. After two generations, tumors were dissociated to single cells and injected intracardially. MRI-detectable macrometastases were observed in 50% and micrometastases in 100% of mice after 8–12 weeks post-injection. The WHIM2 and WHIM5 models were established from tissue from TNBC primary tumour and brain metastases, respectively, from the same patient, and were implanted into the NOD/SCID mice’s mammary fat pads that had been humanized through fibroblast injections [65]. In a later study, xenografts from WHIM2 were cultured in vitro for further expansion and subsequent xenotransplantation via intracardiac injections to generate BCBM [64]. In this model, 100% of mice developed brain metastases, however, animals also developed metastases in the liver (50%), lung (33%), ovaries (83%), and adrenal glands (25%). This study highlighted the importance of studying cancer therapeutics, such as carboplatin and cyclophosphamide, at different metastatic sites, as drug efficacy was shown to vary depending on metastatic location. 

Alternative methods to establish patient-based animal models of BCBM have been described, including those using direct implantation/injection into the animal brain. In order to provide a more direct pathway to disseminating cells within the brain and to extend survival of the experimental model by minimizing metastatic growth elsewhere in the body, a novel protocol of intracarotid injection has been developed, whereby the ligation of the external carotid artery with the retrograde ligation of the common carotid artery during injection of the cancer cells was performed [63]. Dissociated patient-derived BC cells were expanded by intracranial injection to SCID mice and formed metastases were then isolated and transduced with the luciferase gene in vitro. Subsequently, cells were injected into the mouse internal carotid artery, and tumor growth was monitored with BLI. Another model used mice intracranial injections of the tumor cells from the five patients with the HER2+ BCBM to test the targeted therapy combination [66]. PDX models were first generated using intracranial implantation of the patient tissue into the SCID mice. Cancer cells from the xenograft were dissociated, transduced with a luciferase reporter, and then re-injected intracranially into new cohorts of mice to evaluate treatment response using BLI and MRI. The models of intracranial injections of patient-derived cancer cells directly from a patient sample (PDX1435) or from an established PDX (PDX2147) have also been described and used to show that treatment of BCBM by athermal radiofrequency electromagnetic fields at BC specific frequencies (BCF) results in strong suppression and reduction of brain metastasis compared to a sham treatment [32]. Interestingly, using the brain metastasis tumor samples from a TNBC patient, it has been shown that the introduction of tumor samples into the mouse brain directly through a burr hole with a pipette tip results in the 100% engraftment rates compared to using a needle (80%) or forceps (66%) [62]. The pipette method resulted in no mortality of mice throughout the procedure, while the needle method resulted in the mortality of 5/8 mice. Trocars to bilaterally implant tumor fragments subcutaneously from three different brain metastases from BC in mice were also successfully used to establish the BCBM models and to study various PET tracers for metastases detection [67]. It is important to note that intracranial injections and implantations of cancer cells are associated with several major limitations, such as injection-induced BBB disruption and generation of only one single large tumor lesion.

## 3. Detection Methods of BCBM

**Figure 3:**
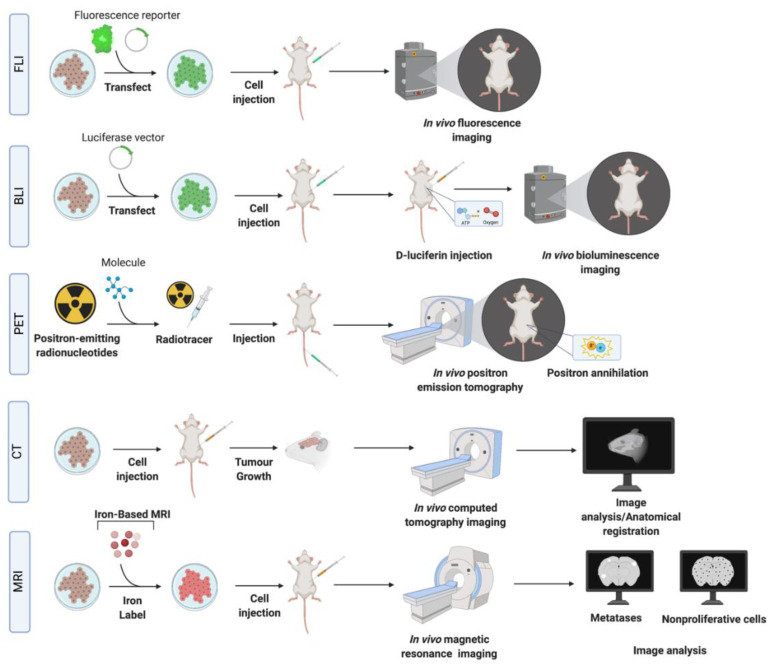
Schematic of in vivo imaging methodologies. Breast cancer brain metastasis models can be imaged with fluorescence imaging (FLI), bioluminescence imaging (BLI), positron emission tomography (PET), computed tomography (CT), and magnetic resonance imaging (MRI) or combinations of these techniques (multimodality imaging) (see text for details). In FLI and BLI, cells are labeled with reporters and introduced into the animal. Substrates are then used to detect a bioluminescent signal in BLI, while no substrates are required for FLI. In PET imaging, radiotracers conjugated to a substrate (see text and Table 2) are used. In targeted PET imaging, radiotracers can be conjugated to antibodies against specific molecules expressed by cancer cells to improve sensitivity and specificity of detection of the metastases. CT and MRI can often use contrast enhancing molecules such as iodine (CT) or gadolinium (MRI) to improve image contrast and detection. MRI can also utilize iron particles (iron-based MRI) to improve cellular detection and allow for monitoring of the arrest, growth, and retention of cancer cells in vivo. Created with BioRender.com.

Histologic evaluation of the brain tissues allows for measurements of the number and size of metastases and their cellular markers. However, these methods are limited by the need to sacrifice the animal with only the endpoint analysis permitted for assessing treatment effects. In vivo imaging methods are the key to a better understanding of the progression of BCBM and dynamic monitoring of tumor responses (Figure 3). A number of imaging modalities have been widely used for the detection of BCBM in experimental models (Table 2). 

**Optical Imaging.** Fluorescence imaging (FLI) and bioluminescence imaging (BLI) techniques (that are based on the detection of light emitted from either fluorescent or bioluminescent reporters, respectively) (Table 2) have been used to study the dissemination and proliferation of cancer cells [81,82]. FLI and BLI signal can be quantified so that the relative amount of light detected can be measured and correlated with the treatment response of a tumor over time [83,84]. Both methods require transfection/transduction of the various types of reporters into cells [85] (Table 2). In FLI, the most commonly utilized reporter, GFP, is used to directly detect the labeled cells through an excitation by a light source without a substrate [86,87]. In BLI, cells expressing reporters, such as luciferase, are detected in the animal through intravenous, intravitreal, or intraperitoneal injection of the substrate. The sensitivity of cell detection with BLI is medium, although higher than FLI [83]. Advances in the development of bioluminescent systems, for example Nanoluc [88] and Akaluc [89], have produced improvements in sensitivity, sometimes down to a single cell detection.

**Computed Tomography (CT).** Micro-CT is applied for the imaging of small animals and has advantages such as low cost, fast acquisition, and high spatial resolution (~100 um) with 3D volumes [90]. The main limitation of micro-CT is related to low contrast, which is overcome by the use of contrast-enhancing agents, such as iodine. The latter technique makes it comparable to MRI in terms of accuracy in determining the volume of BCBM [91,92]. Rapid renal clearance of iodine in mice is a limitation that can be addressed by using “blood-pool” high molecular weight agents, or nanoparticles with slow clearance [93,94] that accumulate in tumors due to the enhanced permeability and retention effect caused by the leaky neovasculature [95] (Table 2). Contrast agents can be modified by conjugating specific ligands to the surface for a targeted imaging approach [96], such as gold nanoparticles linked to the antibodies/proteins for targeting HER2 [97] and other molecules overexpressed on cancer cells [98] and can thus enhance imaging specificity. 

**Positron Emission Tomography (PET).** PET provides an advantage of monitoring changes in tumor metabolism (Table 2). Biological molecules are labeled with isotopes and then introduced into subjects to detect their biodistribution and concentration. Cancer cells have accelerated glycolysis compared to surrounding tissue and this is exploited for PET by using a glucose analogue, fluorodeoxyglucose (FDG), to measure glucose utilization. The utility of FDG for brain metastases is limited because normal brain tissue has a high rate of glucose metabolism and therefore high FDG accumulation [99,100]. Better specificity is achieved by using a fluorescent L-DOPA amino acid analogue FDOPA to capture increased amino acid transport pertinent to cancer cells [101]. Another vulnerability of cancers, hypoxia, can be detected using 18F-fluoromisonidazole. The main limitations of PET imaging for preclinical studies are high background activity, relatively low resolution, and exposure to ionizing radiation [102]. 

**Magnetic Resonance Imaging (MRI).** MRI has been widely used to study BCBM [62,103,104,105] and other brain tumors and its metastases [62,104,105,106]. A variety of types of MRI contrasts generated using different pulse sequences are typically used to highlight the tumor boundary associated edema, necrosis, and hemorrhage. Tumor volumes can be measured and tracked over time. Contrast agents are often employed to enhance the difference in signal between normal and pathological tissues. The most commonly used contrast agent, gadolinium (Gd), does not cross an intact BBB. However, a leaky tumor neovasculature allows Gd penetration, resulting in signal enhancement in post-Gd images. Gd-enhanced MRI has been used in many preclinical cancer models to evaluate BBB permeability associated with brain tumors, and effects of radiotherapy and chemotherapy [47,107].

Another class of MRI contrast agents include superparamagnetic iron oxide (SPIO) nanoparticles loaded into cancer cells by coincubation prior to the injection of cells into an animal. The strong magnetic susceptibility of these iron particles causes a region of signal loss in MR images which is much larger than the size of the cells; a so-called blooming effect. This leads to very high cellular sensitivity and even single SPIO-labeled cells can be detected under optimal conditions [104]. However, iron particles are diluted in the progeny of proliferative cells and therefore labeled cells become undetectable after repeated cell divisions [108]. In contrast to proliferative, non-proliferative cancer cells do not dilute the SPIO and can be identified by MRI as persistent signal voids. The retention of iron in non-proliferative cells was exploited to simultaneously track the fate of both proliferative and non-proliferative cell populations of MDA-MB-231BR cells in the brain [104]. This subpopulation of non-proliferative cancer cells is thought to represent “quiescent” or “dormant” cancer cells (G0-G1 cell cycle arrest) and may proliferate to form metastases in the future. Clinical relevance of this finding is emphasized by studies of whole brain radiotherapy (WBRT) in MDA-MB-231BR-HER2 models [47], where WBRT prevented almost all tumor growth in the brain; however, MRI illustrated persisting signal voids due to non-proliferative, iron-retaining cancer cells over time. These results are in line with other preclinical studies that suggest quiescent cells are not responsive to cancer chemotherapies designed to target proliferating cells [109,110].

**Multimodality Imaging.** Multiple imaging modalities are often used in a complementary way to acquire multi-layered information, combining advantages of each individual modality. The development of hybrid imaging systems has advanced multimodality imaging by allowing multiple types of images to be obtained in the same scanner without moving the subject. For example, images acquired with MRI or CT are often combined with PET, providing anatomical, functional, and metabolic information in one session. Combinations of SPIO MRI with PET/CT [111] and BLI [112] and PET/CT/BLI have been used to evaluate brain metastases and monitor progression beyond the brain and to evaluate treatment responses. Multimodality imaging can also allow targeted imaging approaches, such as targeted PET imaging using F-18-labeled HER2 reactive antibodies [113]. Using a targeted PET system with CT and BLI in athymic nu/nu mice intracranially implanted with BT474M1BrM3-Fluc cells [113], brain metastases could be visualized after intravenous administration of F-18-labeled anti-HER2 antibodies. CT was used for anatomical localization of the PET signal and BLI to monitor tumor growth. 

## 4. Discussion and Conclusions

Substantial advances in preclinical BCBM models and their detection have been recently made. Efficient formation of BCBM depends on the cancer cell model, its molecular profile, method of introduction of cancer cells, and their selection for metastasizing in the brain. An additional challenge in developing successful preclinical experimental models is the significant molecular heterogeneity of BC that represents a compilation of various malignant entities [114]. A majority of the models are based on the use of the IBCC lines propagated through numerous passages, decreasing their utility to represent BC heterogeneity, unique characteristics of brain metastases, and our ability to draw generalizations for clinical applications [115,116]. Moreover, IBCC lines are originally derived from non-brain metastatic sites (Table 1) and, as such, may not accurately represent the biology and metastatic behaviour of the brain metastatic disease they are intended to model [117]. To enhance the specificity of the development of metastases in the brain, various approaches were used to establish brain-seeking cell lines that require rigorous selection and numerous passages leading to potential selection bottlenecks contributing to the further loss of heterogeneity and selection biases [118]; all of which are undesirable factors for generalizations of treatment responses. 

Recent advances in growing patient-derived BC cells creates the potential to address these shortcomings [119,120]. In order to better capture BC heterogeneity and treatment responses observed in individual patients, patient-derived organoids (PDO) and PDX systems have been developed [74,121,122]. Several animal BCBM models have now been described that utilize patient-derived cancer tissues, including those from patient’s BCBM. These approaches make studies of treatment of BCBM more relevant to the clinical setting. However, generation of metastases by PDX models is relatively challenging. Most of the models use implantation of the original patient cancer tissue into a mouse to generate PDX and expand cancer cells. The tissue from the xenograft is then used to generate the BCBM model, sometimes requiring cycles of re-passaging in vivo and/or in vitro to generate an efficient model. Passaging of PDX tissue in vitro (for expansion or introduction of reporters) often leads to the contamination of the culture by mouse cells, which can represent a significant experimental drawback. Few studies have accounted for the presence of mouse cell contamination from the PDX tissue in the in vitro culture system. In such situations, the selection of human cells is required that might lead to additional losses in the heterogeneity of the sample. An alternative to the aforementioned approach could be based on PDO, which are used for the expansion of tumor cells in vitro from the patient’s original sample and are devoid of contamination by the mouse cells. The PDO model system allows long-term expansion and genetic manipulation (such as reporter transduction) of patient-derived cancer cells in vitro. Expanded and/or transduced PDOs can then be introduced into the mice (PDO xenografts or PDOX) to generate BCBM. The PDO-PDOX approach to study BCBM metastases is an alternative promising approach for generating efficient BCBM preclinical models that have not yet been exploited. Recently, Cosgrove et al. demonstrated that freshly resected BCBM tumours could be used to generate PDO and perform genomic and transcriptomic analysis to identify therapeutic vulnerabilities [123]. 

Finally, most of the models are based on the utilization of immune-compromised mice and thus lack the immune components of the tumor microenvironment that are essential for tumor colonization and progression [68,124]. The importance of the tumor microenvironment in the brain can also be gauged from the fact that the same cancer cell line may exhibit differential growth characteristics depending on the strain of the mouse used for cell line establishment [125]. These differences could be explained by the differences in immune competency of each strain, such as the lack of T cells in nude mice compared to the lack of T, NK, and B cells and defective macrophages and dendritic cells in the severely immune compromised NSG mouse. To overcome those limitations, syngeneic models are used. While these models do account for the immune responses and tumor microenvironment by utilizing immune competent mice, it is important to consider that the cell lines are not of human origin. On the other hand, the development of humanized models [64,65] created an opportunity to use BC from human tissues combined with the advantage of the presence of the components of the immune system within the animal model. 

Historically, the most extensively used models of BCBM were those based on immortalized cell lines, particularly MDA-MB-231BR and MDA-MB-231BR-HER2+ introduced to mice via intracardiac injections. Overall, the main challenges associated with BCBM models based on immortalized cell lines include multiple passaging and clonal selections that might lead to genetic bottlenecks, lack of tumour heterogeneity, and derivation from sites other than the brain, thus reducing their potential for translational research. However, compared to PDO/PDX, they are relatively less expensive, and easier to culture and perform genetic manipulations on. PDO/PDX models are more costly, labour-intensive and more difficult to establish. These disadvantages of PDO/PDX models however are outweighed by the fact that they better represent the genetic characteristics and heterogeneity of the individual patient’s tumour, as well as better mimic responses to treatment observed in individual patients. Due to those factors, the use of patient-derived models for studies of BCBM is gaining momentum, especially for translational drug and radiotherapy studies. Last, but not least, these models lack a tumour microenvironment, limiting their applications in terms of immunotherapy studies. For the latter, syngeneic models can be utilized; however, they are based on murine breast cancer cells and might not recapitulate nuances of pathology of human disease. Developments in humanized mouse models might open new avenues for preclinical immunotherapy studies in BCBM. 

Sensitive and specific experimental animal imaging techniques for detection and monitoring BCBM is one of the keys for studying preclinical BCBM models and assessing treatment responses. The most common BCBM detection techniques have been based on MRI, BLI, and histological assessment of the tissue. Advances in preclinical imaging, such as cellular MRI, targeted imaging, and the use of multimodality imaging, provide an opportunity for further improvement of the preclinical BCBM models. The choice of imaging modality depends on the spatial resolution, specificity, and sensitivity required for detection, the depth of the metastases being imaged and the potential for clinical translation. Further advances in preclinical imaging of BCBM are expected, with improvements to image resolution and sensitivity, more specific and/or targeted contrast agents and more widespread implementation of multimodality imaging approaches.

## Figures and Tables

**Table 1:** Preclinical models for studies of breast cancer brain metastases.

	Cell Type	Origin	Subtype	Animal Model	Injection Method	Detection Method	Drugs Studied	Original Reference
**Xenogeneic**	MDA-MB-361	Brain metastasis	ER+/PR+/HER2+	Nude mice	Intracarotid	Histology	Docetaxel, doxorubicin and cyclophosphamide [20]	[21]
	MDA-MB-468	Pleural effusion	TNBC	Nude mice	Intracarotid	Histology	Docetaxel [22]	[21]
	MA11	Bone marrow aspirate	TNBC	BALB/C nu/nu nude mice	Intracardiac	Autopsy, Histology, and MRI	Ionizing radiation and trichostatin A (HDAC inhibitor) [23]	[24]
	MDA-MB-231BR	Pleural effusion	TNBC	Nude mice	Intracardiac	Histology	Vorinostat [25] DAPT [26] GSK461364A [27] HA-paclitaxel nanoconjugate [28] Saracatinib with lapatinib [29] Whole brain radiotherapy [30,31] BCF [32] ANG1005/GRN1005 [33] iRGD nanoparticles [34] Azacitidine [35] WP1066 [36] Radiation with ultrasound-ruptured oxygen microbubbles [37] mTOR inhibitors (rapamycin, Temsirolimus-CCI-779) [38]	[39]
	MDA-MB-231BR1, -BR2, -BR3	Pleural effusion	TNBC	Athymic NCr-nu/nu mice	Intracarotid	Histology	PTK787/Z 222584 [40] Temozolomide [41]	[40]
	MDA-MB- 231-BrM2	Pleural effusion	TNBC	Athymic nude mice	Intracardiac	BLI, MRI, Histology	GDC-0068 [42]	[43]
	MDA-MB-231BR-HER2+	Pleural effusion, then brain metastases in mice	ER-/PR-/HER2+	BALB/c nude mice	Intracardiac	Immunofluorescence	Lapatinib [44] Pazopanib [45] LRRC31 nanoparticles with radiation [46] Whole brain radiotherapy [47]	[48]
	CN34-BrM2	Pleural effusion	TNBC	Beige nude mice	Intracardiac	BLI, MRI, Histology	mTOR inhibitors (rapamycin, Temsirolimus-CCI-779) [38]	[43]
	JIMT-1-BR3	Pleural effusion	HER2+	NRC nu/nu mice	Intracardiac	Histology	Temozolomide [41]	[41]
	SUM190-BR3	Primary tumor	HER2+	Athymic NIH nu/nu mice	Intracardiac	Immunofluorescence	N/A	[49]
	BT474.br/Br.2/Br.3	Primary tumor	ER+/PR+/HER2+	Swiss nude mice	Intracarotid	Confocal microscopy, Immunofluorescence	Vardenafil and trastuzumab [50] Lapatinib and trastuzumab [51] TAK-285 [52] Saracatinib with lapatinib [29]	[29]
	SKBrM3+	Plural effusion	ER-/PR-/HER2+	Athymic nude mice	Mammary fat pad	BLI, Histology	Cabozantinib and Neratinib [53]	[53]
**Syngeneic**	Br7-C5	N-ethyl-N nitrosourea-induced mammary adenocarci- noma	Unspecified	Berlin–Druckrey IV rat	Intracardiac	Histology	N/A	[54]
	4T1BM	Murine mammary carcinoma	TNBC	Syngeneic BALB/c mice	Mammary fat pad	Histology	N/A	[55]
	4T1Br4	Murine mammary carcinoma	TNBC	Syngeneic BALB/c mice	Mammary fat pad	Histology	Trebananib [56]	[57]
	4T1-Luc	Murine mammary carcinoma	TNBC	Syngeneic BALB/c mice	Intracranial, intracardiac, spontaneous	BLI	Fluphenazine hydrochloride [58]	[59]
	TBCP-1	Spontaneous BALB/C mammary tumor	ER-/PR-/HER2+	Syngeneic BALB/C mice	Intracardiac	Histology	Neratinib [60]	[60]
**Patient-Derived**	F2-7	Patient brain metastases	TNBC	NSG mice	Intracardiac	BLI	N/A	[61]
	Brain-orthotopic PDXs	Patient brain metastases	TNBC and ER+ varied	NSG mice	Intracranial (pipette method)	Histology	N/A	[62]
	BM-E22-1	Patient brain metastases	TNBC	NSG mice	Intracardiac	MRI	N/A	[61]
	DF-BM#Ni7, DF-BM#656	Patient brain metastases	ER+ HER2+ (DF-BM#Ni7), TNBC (DF-BM#656)	NOD/SCID mice	Intracarotid (ligation method)	BLI	N/A	[63]
	WHIM 2/WHIM5	Primary tumor/patient brain metastases	TNBC	NOD/SCID mice	Mammary fat pad	Histology	Carboplatin, cyclophosphamide, bortezomib, dacarbazine [64]	[65]
	PDX1435/PDX2147	Patient brain metastases (PDX1435), primary tumor (PDX 2147)	TNBC	NOD/SCID mice	Intracranial	MRI	BCF [32]	[32]
	Orthotopic HER2+ PDXs	Patient brain metastases	HER2+, ER/PR status varied	NOD/SCID mice	Intracranial	BLI, MRI	Combination of PI3K inhibitor (BKM120) and mTORC1 inhibitor (RAD001) [66]	[66]
	Subcutaneous PDXs	Patient brain metastases	Unspecified	SCID BALB/c mice	Subcutaneous (trocar method)	PET/CT	N/A	[67]

Abbreviations: Bagg Albino (BALB), bioluminescence imaging (BLI), breast cancer specific frequencies (BCF), computerized tomography (CT), dual antiplatelet therapy (DAPT), estrogen receptor (ER), histone deacetylases (HDAC), human epidermal growth factor receptor 2 (HER2), magnetic resonance imaging (MRI), mechanistic target of rapamycin (mTOR), NOD/SCID/Gamma (NSG), nonobese diabetic/severe combined immunodeficiency (NOD/SCID), positron emission tomography (PET), progesterone receptor (PR), severe combined immunodeficiency (SCID), triple negative breast cancer (TNBC).

**Table 2:** Imaging modalities used for detection of BCBM in animal models.

Imaging Modality	Principles	Reporters /Detection Used	SR/S/HS/Sp	Information	Advantages	Disadvantages and Limitations for Imaging
**BLI**	Optical detection of light emitted from BLI reporters.	Genetically expressed proteins such as luciferase	SR—~1 mm S—Medium (1000 s of cells) HS—one cell Sp—High	Probe uptake, cell presence, and cell viability.	Minimally invasive, inexpensive, allows for signal quantification, whole mouse imaging and has high throughput. BLI signal is only produced by viable cancer cells permitting distinction between viable and dead cells.	Requires stable transfection of the reporter into cancer cells and injection of substrate into a mouse ^a^. Limited depth penetration and therefore, not clinically translatable. Challenging to determine depth of a tumor within the body based on the signal. False negative effects can occur in areas where the substrate cannot easily accumulate, such as the brain, or in tumors with compromised vasculature. Probe uptake in the brain and limited imaging depth in biological tissues.
**FLI**	Optical detection of light emitted from fluorescent reporters.	GFP, eGFP, EYFP, mCherry, TagRFP, Dendra2, tdTomato.	SR—~1 mm S—Medium Sp—High	Probe uptake, cell presence and cell viability.	Minimally invasive, inexpensive, allows for whole mouse imaging and has high throughput. Does not require injection of substrate. The signal is quantifiable.	Requires stable transfection/transduction of the reporter into cancer cells and excitation by an external light source. Background autofluoresence decreases sensitivity. Challenging to determine depth of a tumor within the body based on the signal. Probe uptake in the brain and limited imaging depth in biological tissues.
**CT (with and without contrast)**	Combinations of multiple X-ray measurements taken from different angles to produce tomographic images. With a contrast agent, CT images can reveal the location and density of vessels (early), and contrast agent accumulation in the tissue (late).	Iodine-containing polymers [77], liposomes [78] or micelles [79] and gold nanoparticles [80].	SR—~ 100 um S—Low Sp—Medium	Tomographic images, vessel density, and agent accumulation.	Low cost, fast acquisition and high spatial resolution of 3D volumes.	Radiation exposure, low contrast can make certain pathologies difficult to discern; contrast-enhanced micro-CT is more commonly applied. Low contrast does not allow for visualization of tumor detail, often needs contrast enhancement.
**PET**	Detection of γ rays from positron emitting radioisotopes ^b^.	FDG, ^18^F-FMISO.	SR—~1 mm S—High picomolar (100–1000 s of cells) Sp—High	Tracer uptake; biological and biochemical. Direct cell quantification, and signal specific to cells.	Can monitor tissue metabolism (glycolysis, DNA synthesis, amino acid transport and oxygenation state) in brain metastases, with excellent depth penetration.	Requires tracers, normal brain tissue has a high rate of glucose metabolism and therefore high FDG accumulation which decreases specificity. Signal decays over time (t_1/2_), and cells are exposed to radioactivity. Low radiotracer uptake in brain.
**MRI (proton)**	Detection of water proton relaxation after RF absorption.	See below.	SR—500–2000 microns S—Low millimolar Sp—Medium	Anatomical information, morphology, and tissue composition.	No ionizing radiation exposure, provides excellent soft tissue contrast.	Potential tissue heating during long scans, risk of peripheral nerve stimulation, sensitive to motion. Poor sensitivity in detecting micrometastases.
**MRI (contrast)**	MRI with use of contrast agents, administered to improve signal differences between normal and cancerous tissue.	Most common contrasts—gadolinium-based, manganese-based.	SR—500–2000 microns S—medium Sp—Medium	Improved visibility of tumors, inflammation, and blood supply.	No radiation exposure. Clinically, dynamic contrast enhanced (DCE) MRI can be used to image the tumor vasculature by acquiring sequential images during the passage of gadolinium through tissues and provides quantitative measures of perfusion, permeability and blood volume.	Requires administration of contrast. Heterogeneity of metastasis permeability in early and late stages of development.
**MRI (iron nanoparticles)**	Detection of intracellular iron particles via distortion of the magnetic field.	SPIO nanoparticles labeling via co-incubation with cancer cells.	SR—200–1000 microns S—High picomolar HS—one cell Sp—Medium	Cell location and presence, including nonproliferative cells.	High sensitivity, non-proliferative, cancer cells do not dilute the SPIO and can be identified by MRI as persistent signal voids by virtue of their retaining iron.	SPIO are diluted in the progeny of proliferative cells and therefore labeled cells become undetectable by MRI after repeated cell divisions. Poor cell quantification. Other structures in brain appear with low signal (i.e., blood, air, bone).

^a^ For bacteria that produce their own substrate no injection is required. ^b^ Frequently used isotopes include fluorine (18F), copper (64Cu), carbon (11C), nitrogen (13N) and oxygen (14O). Abbreviations: 18F-fluoromisonidazole (^18^F-FMISO), bioluminescence imaging (BLI), 2-[18F]fluoro-2-deoxy-D-glucose (FDG), green fluorescent protein (GFP), computed tomography (CT), dynamic contrast enhanced (DCE), enhanced green fluorescent protein (eGFP), enhanced yellow fluorescent protein (eYFP), fluorescence imaging (FLI), highest sensitivity reported (HS), magnetic resonance imaging (MRI), positron emission tomography (PET), sensitivity (S), spatial resolution (SR), specificity (Sp), superparamagnetic iron oxide particles (SPIO), tag red fluorescent protein (TagRFP), tandem dimer tomato (tdTomato).

## Data Availability

Not applicable.

## Abbreviations

231BR	MDA-MB-231BR
BBB	blood–brain barrier
BCBM	breast cancer brain metastasis
BCF	breast cancer specific frequencies
BLI	bioluminescence imaging
BC	breast cancer
CT	computed tomography
ER	estrogen receptor
FDG	fluorodeoxyglucose
FLI	fluorescence imaging
Gd	gadolinium
HER2	human epidermal growth factor receptor 2
IBCC	human immortalized breast cancer cell
MRI	magnetic resonance imaging
PDO	patient-derived organoids
PDOX	patient-derived organoid xenografts
PDX	patient-derived xenografts
PET	positron emission tomography
PR	progesterone receptor
SPIO	superparamagnetic iron oxide particles
TNBC	triple negative breast cancer
VEGF-A	vascular endothelial growth factor A
WBRT	whole brain radiotherapy
eGFP	enhanced green fluorescent protein
